# Evolution of the Maillard Reaction in Glutamine or Arginine-Dextrinomaltose Model Systems

**DOI:** 10.3390/foods5040086

**Published:** 2016-12-07

**Authors:** Silvia Pastoriza, José Ángel Rufián-Henares, Belén García-Villanova, Eduardo Guerra-Hernández

**Affiliations:** 1 Departamento de Nutrición y Bromatología, Facultad de Farmacia, Universidad de Granada, Campus Universitario de Cartuja, 18071 Granada, Spain; spdelacueva@ugr.es (S.P.); belenv@ugr.es (B.G.-V.); ejguerra@ugr.es (E.G.-H.); 2 Departamento de Nutrición y Bromatología, Instituto de Investigación Biosanitariaibs.GRANADA, Universidad de Granada, 18071 Granada, Spain

**Keywords:** glutamine, arginine, dextrinomaltose, model systems, Maillard reaction, enteral formulas

## Abstract

Enteral formulas are foods designed for medical uses to feed patients who are unable to eat normally. They are prepared by mixing proteins, amino acids, carbohydrates and fats and submitted to sterilization. During thermal treatment, the Maillard reaction takes place through the reaction of animo acids with reducing sugars. Thus, although glutamine and arginine are usually added to improve the nutritional value of enteral formulas, their final concentration may vary. Thus, in the present paper the early, intermediate, and advanced states of the Maillard reaction were studied in model systems by measuring loss of free amino acids through the decrease of fluorescence intensity with *o*-phtaldialdehyde (OPA), 5-Hydroximethylfurfural (HMF), furfural, glucosylisomaltol, fluorescence, and absorbance at 420 nm. The systems were prepared by mixing glutamine or arginine with dextrinomaltose (similar ingredients to those used in special enteral formula), and heated at 100 °C, 120 °C and 140 °C for 0 to 30 min. The recorded changes in the concentration of furanic compounds was only useful for longer heating times of high temperatures, while absorbance and fluorescence measurements were useful in all the assayed conditions. In addition, easiness and sensitivity of absorbance and fluorescence make them useful techniques that could be implemented as indicators for monitoring the manufacture of special enteral formulas. Glucosylisomaltol is a useful indicator to monitor the manufacture of glutamine-enriched enteral formulas.

## 1. Introduction

Enteral formulas are the most common products used to feed patients who are unable or unwilling to eat normally. They are prepared by mixing proteins, carbohydrates, fats and other nutrients [1,2]. Although milk proteins are the main nitrogen source in enteral formulas, some patients have specific nutritional needs that are met by special enteral formulas with added free amino acids. One of these added amino acids is glutamine, a non-essential amino acid that becomes essential in critically ill patients with metabolic stress [3], to stimulate intestinal functionality [4,5] and the immune response [6]. Likewise, arginine is another amino acid that is essential to patients with metabolic stress [7]. Broadly speaking, arginine improves nitrogen uptake, increases collagen synthesis, and stimulates the immunity response, especially cell-mediated immunity [8].

Enteral formulas can be altered by the heat treatment used to sterilize them [9] or by their storage conditions [10], including temperature and light exposure. The Maillard reaction (MR) is one of the main chemical reactions in many foods, such as dairy products, giving rise to a decrease in nutritional quality, especially loss of lysine [11]. In the early stage the free amino groups of proteins or free amino acids react non-enzymatically with reducing sugars, giving rise to the subsequent formation of Amadori compounds. Then, in the intermediate stage different compounds are formed, such as furanic compounds. Finally, in the advanced step of the reaction, intermediate Maillard reaction compounds are re-arranged into colored, fluorescent and cross-linked molecules such as melanoidins [12].

Early stages of Maillard reaction can be evaluated by measuring the loss of fluorescence intensity with *o*-phthaldialdehyde (OPA), which gives information about the blockage of amino acids, especially lysine [9,10,13]. In addition, Maillard-mediated reaction leads to the oxidative deamination of lysine residues to yield carbonyl compounds in proteins like α-aminoadipic and γ-glutamic semialdehydes, which in turn are different from dicarbonyls formed from oxidized sugars [14]. Intermediate stages of Maillard reaction can be followed in milk and milk-derived products by the classic measurement of furanic compounds such us 5-Hydroxymethylfurfural (HMF), furfural [15,16]. Another useful indicator for the intermediate stages is glucosylisomaltol, which is formed during the heating of maltose with primary or secondary amines [17] and has been used to control heat treatment in baby cereals [18,19]. Finally, the advanced stages of the MR can be studied by the measurement of fluorescent [12,20,21] and colored compounds [12,22]. Thus, there is a linear correlation between the time/temperature of heat treatment and the fluorescence and color detected [21], being useful indicators to evaluate the efficiency of technological industrial processes in enteral formulas [23].

Because of the complexity of Maillard reaction, most published studies have been conducted with model systems. Some of these models cannot be directly applied to liquid milk-resembling systems (enteral formulas) because of the different reaction conditions (water activity, moisture, pH, etc.) and the reactivity of proteins (caseins, whey proteins) and reducing sugars (dextrinomaltose, lactose). The present study was designed to study the extent of the MR in its early, intermediate and advanced stages in heated dextrinomaltose-amino acids solutions (glutamine and arginine) with similar heat treatment and concentrations to enteral formulas. Thus, the study will allow the selection of the best indicators to control amino acids blockage through the rearrangement derived from the Maillard reaction during heat treatment without the possible interferences derived from other constituents of enteral formulas.

## 2. Materials and Methods

### 2.1. Samples

The amino acids glutamine and arginine were purchased from Sigma-Aldrich (Barcelona, Spain) and dextrinomaltose was supplied by a Spanish dietetic product company. Model systems were performed by dissolving (at concentrations similar to those of enteral formulas) glutamine or arginine (4% *w*/*v*) and dextrinomaltose (12% *w*/*v*) in 100 mL of 0.1 M phosphate buffer at pH 6.5. Two aliquots of 10 mL were then placed in Pyrex screw-cap vials, which were immersed in a glycerol bath kept at 100 °C, 120 °C and 140 °C for 0, 5, 10, 15, 20, 25 and 30 min. The samples were then cooled in an ice bath and stored at −50 °C until their analysis. The heating times reported here exclude the heating-up period, which was estimated at 2 min.

### 2.2. Analytical Methods

Six different indicators were assayed in order to study the evolution of the Maillard reaction.

**OPA** determination was assayed by measuring the loss of fluorescence intensity derived from the reaction between OPA and glutamine or arginine [9]. Sodium dodecyl sulphate (SDS) (1.5 mL) 12% (*w*/*v*) solution was added to 1.5 mL of diluted sample (containing 2 mg·mL^−1^ of amino acid) and then refrigerated overnight. Then, 1 mL was mixed with 2 mL of water and 2 mL of freshly prepared OPA reagent (16.4 mg in 2.5 mL of 95% methanol, 5 mL of 20% SDS, 25 mL of 0.1 M borate buffer pH 9.5, 400 μL of 10% β-mercaptoethanol solution made up to 100 mL with distilled water) with constant stirring, incubated for 2 min at 25 °C, and the relative fluorescence (RF) was measured after 3 min at emission and excitation wavelengths of 455 nm and 340 nm, respectively, with a fluorescence spectrophotometer (Perkin-Elmer, mod. 204, Waltham, MA, USA). Quinine sulfate solution (5 mg·mL^−1^ in 0.1 N H_2_SO_4_) was prepared daily as 100% RF calibration standard.

**HMF**, **furfural** and **glucosylisomaltol** (GIM) were determined by HPLC after clarification with Carrez I and II reagents [16,19]. Briefly, 2 mL of sample were mixed suspended in 5 mL of deionised water in a 10 mL centrifuge tube. The mixture was clarified with 0.25 mL each of potassium ferrocyanide (15% *w*/*v*) and zinc acetate (30% *w*/*v*) solutions and centrifuged at 4500 *g* for 10 min at 4 °C. The supernatant was collected in a 10 mL volumetric flask made up to 10 mL with deionised water. Then 500 μL were filtered through 0.45 μM filter and injected on an Accela 600 HPLC (Thermo-Scientific, Waltham, MA, USA). The mobile phase was a mixture of acetonitrile in water (5% *v*/*v*) delivered at a flow rate of 1 mL·min^−1^ under isocratic conditions through the analytical column (Extrasyl-ODS2, 25 × 0.40 cm, 5 mm particle size, Tecknokroma, Barcelona, Spain) thermostated at 32 °C. The UV detector was set at 284, 277, and 280 nm for HMF, furfural and GIM, respectively, and 20 µL of the extract were injected.

**Color** was determined as absorbance at 420 and 600 nm of the extracts obtained for HMF analysis, subtracting the latter from the former to obtain the turbidity. The unheated systems were used as blank [16,22].

**Fluorescence** determination was done by measuring the fluorescence intensity at λ_ex_ = 345 nm and λ_em_ = 415 nm of 500 μL of the model system diluted with phosphate buffer [24]. The fluorescence was set up at 100% with a quinine sulphate standard.

**Complementary analyses**: The content of mono- di- and trisaccharides were determined by gas chromatography (GC) as trimethylsilyl oximes [25].

### 2.3. Statistical Analysis

Data were reported as means ± standard deviations. Each experiment was performed in triplicate. The Student’s test was used to compare means (significance level *p* < 0.05) and was conducted by using SPSS software (version 23, IBM Corp., Armonk, NY, USA). Person lineal correlation was studied in order to correlate HMF and GIM data.

## 3. Results and Discussion

The extent of the Maillard reaction was studied during the early (OPA fluorescence), intermediate (HMF, furfural and glucosylisomaltol) and advanced (absorbance and fluorescence) stages. The model system studied contained dextrinomaltose, whose proximal composition revealed a content of 4.25%, 4.30% and 4.60% (*w*/*w*) of glucose, maltose, and maltotriose, respectively. These mono- di- and trisaccharides have reducing activity, giving rise to the development of the Maillard reaction in model systems [22,24]. In fact, other authors reported in liquid milk-derivatives the effect of dextrinomaltose with different dextrose equivalents on lysine blockage through the Maillard reaction [26].

### 3.1. OPA Fluorescence

*O*-phtaldialdehyde reacts with the amino group of an amino acid forming a quantifiable fluorescent compound [13]. Therefore, the loss of fluorescence intensity can follow the first stages of the Maillard reaction due to the decrease of amino acids availability through the reaction with carbonyl compounds. Table 1 shows the results obtained in the study of model systems heated at 120 °C (usual temperature of enteral formula sterilization). Both amino acids showed an exponential loss of fluorescence intensity with OPA (*p* < 0.05) that correlated with a high reaction rate, always greater in the arginine-dextrinomaltose model system. Similar results were obtained by Lamberts et al. [27] in model systems, who found a higher decrease of arginine compared to other free amino acids (including glutamine). This could be related with the experimental conditions used since arginine is an intermediate reactive amino acid [27] but other authors report increased reactivity of arginine when heated under different conditions [28,29]. After 20 min (usual time for in-bottle enteral formula sterilization) a plateau was reached, including an 85%–90% of fluorescence loss for glutamine and arginine, respectively. These losses are four times higher to those found during sterilization of regular enteral formulas [9]. This difference could be related with the higher availability of amino groups in model systems compared to those found in regular enteral formulas, which are formulated with milk proteins and not enriched with free amino acids.

### 3.2. HMF

Table 2 shows the HMF evolution during the heating of model systems at 100 °C, 120 °C and 140 °C for 30 min. In the glutamine-dextrinomaltose model system, HMF was significantly (*p* < 0.05) increased versus baseline levels only after 30 min at 100 °C, 20 min at 120 °C and 10 min at 140 °C. HMF levels at time 0 are derived from dextrinomaltose, which provides HMF generated from caramelization during dextrinomaltose production [16]. Differences among HMF values were also statistically different (*p* < 0.05) for the same heating time at the three temperatures assessed. In addition, an induction phase was observed for HMF generation. Thus, 10 min of heating at 140 °C was required before changes in its concentration were detected, which is in agreement with other authors [12]; As stated above for loss of fluorescence with OPA, the arginine-dextrinomaltose model system was more reactive, being HMF generated after only 5 min of heating at all assayed temperatures (thus, no induction period was observed). HMF levels increased significantly (*p* < 0.05) with time and temperature of heating.

The dextrinomaltose concentration of the two model systems was similar. In addition, HMF levels did not increase at 100 °C in the glutamine-dextrinomaltose model system while HMF was generated in the arginine-dextrinomaltose model. Therefore, in could be hypothesized that, at least at mild sterilization temperatures (100 °C), HMF derives from the Maillard reaction and not from dextrinomaltose degradation. Indeed, such degradation occurs through caramelization, which accounts at temperatures higher than 150 °C [30].

In general, the HMF levels found in both model systems are lower to those reported for enteral formulas [15], which could be related with the use of a commercial dextrinomaltose with low HMF levels. Only the HMF content of arginine-dextrinomaltose solutions heated for 25–30 min at 140 °C were similar to those of enteral formulas. Given that enteral formulas are sterilized at approximately 120 °C for 20 min or less [9], HMF may be a useful indicator for formulas containing arginine because of the higher rate constants; i.e., for the same temperatures and heating times, more HMF is generated in model systems with arginine. Thus, HMF determination could be of interest in arginine-enriched enteral formulas but not in glutamine-enriched ones (in that case it could not be differentiated between dextrinomaltose-derived HMF and that generated from the reaction glutamine-dextrinomaltose).

### 3.3. Furfural

The utility of furfural as a browning indicator in model systems was studied under similar conditions to those described above for HMF. In glutamine-dextrinomaltose model systems an induction period was observed, being furfural generated after 30 min of heating at 100 °C, 20 min at 120 °C, and after 10 min at 140 °C (Table 3). Furfural levels also increased significantly (*p* < 0.05) with heating time. On the other hand, in the arginine-dextrinomaltose model system the induction period was shorter, being furfural detected after 20 min of heating at 100 °C and after 5 min of heating at 120 °C or 140 °C (Table 3). Furfural values significantly differed among all times and temperatures. These levels are in line with those reported for enteral formulas [16] but lower for those stated in the case of liquid infant formulas [15]. This could be related with the presence of lactose in infant formulas, whose reducing capability is higher than that of dextrinomaltose, being then more reactive through the Maillard reaction.

Furfural, like HMF, may be a useful indicator in enteral formulas prepared with arginine since their sterilization temperature is approximately 120 °C for no more than 20 min. Furfural has the advantage that it is not found in dextrinomaltose but the drawback is that only small amounts are generated.

### 3.4. Glucosylisomaltol

Glucosylisomaltol (GIM) is a furanic compound produced exclusively through the Maillard reaction of maltose and glutamine [31]. Thus, GIM has been proposed as useful indicator to control the manufacture and storage of pasta [31], breakfast cereals [19], baby cereals and bread [18]. Table 4 shows the glucosylisomaltol content in the glutamine-dextrinomaltose model system. No GIM was found in arginine-dextrinomaltose model systems because of the necessity of glutamine for GIM generation [31]. In the glutamine-dextrinomaltose system, glucosylisomaltol was generated after 25 min of heating at 100 °C, 15 min at 120 °C and after 10 min for 140 °C (*p* < 0.05). The values obtained at the different temperatures were also significantly different. In this sense, the generation of glucosylisomaltol showed a similar induction period to that observed for other furanic compounds. Thus, a linear correlation between GIM and HMF contents at all the assayed temperatures was found (*r* = 0.998; *p* < 0.05).

Glucosylisomaltol may be a useful indicator in enteral formulas supplemented with glutamine due to the presence of dextrinomaltose. It is generated at lower times and temperatures compared with other furanic compounds generation (HMF and furfural), so that it may be a more useful indicator to control the heat treatment of enteral formulas. Moreover, unlike HMF, it is not found among the ingredients of enteral formulas (dextrinomaltose, glucose syrup) [16]. In this way, two special enteral formulas enriched with a 2% of glutamine were analyzed for glucosylisomaltol presence (data not shown) ranging the concentration between 0.24 and 0.41 mg·L^−1^. These results are in the same range of the time-temperature used to sterilize enteral formulas (0.59 mg·L^−1^ at 120 °C for 20 min).

### 3.5. Color

The Maillard reaction ends with the production of colored compounds such as melanoidins, giving rise to browning development, that can be found by measuring the absorbance at 420 nm [28]. The results obtained about absorbance evolution in both model systems are depicted in Table 5. Absorbance increased at all the times and temperatures assayed, being statistically different (*p* < 0.05) in all cases. No induction period was observed for color development, which is in agreement with Niquet and Tessier [12]. In the case of glutamine model systems, the results obtained at 140 °C in line with those reported by other authors [12]. Absorbance values were higher in model systems with arginine, which correlates with the higher reactivity of arginine in the conditions used along the study. Thus, at 140 °C there was no increase in absorbance after 15 min of heating, which could be related with the formation of insoluble colored compounds in the last steps of the Maillard reaction. Then, in special enteral formulas with some of these amino acids added as free salts, the determination of absorbance at 420 nm may be a sensitive measurement for controlling heat treatment in the industrial environment.

### 3.6. Fluorescence

A comparative study of nonenzymatic browning in heated glutamine- and arginine-dextrinomaltose solutions and formation of fluorophores was also carried out. The fluorescence intensity significantly differed among all the times and temperatures assayed (Table 6). The fluorescence intensity of arginine model systems was double to that observed for glutamine ones (*p* < 0.05). Under our conditions, the formation of the fluorophores did not exhibit an induction phase, in consonance with the findings reported by Morales et al. [20]. This could be explained taking into account that colorless chemical species (fluorophores) are usually precursors of brown pigments. Since browning did not show an induction period, it is logical that those precursors of brownish compounds also have the same behavior. Contrary to color development, fluorescence still increased in arginine model systems heated at 140 °C. Thus, fluorescence analysis could be of interest to control the manufacture of special enteral formulas.

## 4. Conclusions

The results of this study revealed that the reactivity of arginine was higher than that of glutamine solutions. At sterilization temperatures (100–120 °C) all the indicators assayed, with the exception of glucosylisomaltol, may be useful to control the thermal stress in arginine-dextrinomaltose model systems. For glutamine-dextrinomaltose, only HMF and furfural were useless. Glucosylisomaltol was only detected in glutamine model systems. It was more sensitive to time-temperature increase than furanic compounds and was not detected in enteral formulas ingredients. Thus, glucosylisomaltol may be a useful indicator to monitor the sterilization process of special enteral formulas enriched with glutamine (in fact, it was detected in the two glutamine-enriched enteral formulas analyzed). It must be pointed out that absorbance and fluorescence measurements could be the best indicators for monitoring the manufacture of special enteral formulas since they are fast, sensitive and they do not need state-of-the-art equipment.

Finally, from a hygienic point of view, the usual [9] sterilization temperatures of enteral formulas (120 °C for up to 20 min) assure the microbial stability of the products, although decreasing the sterilization time up to 10 min could be optimal under the nutritional point of view by reducing the decrease of the nutritional value and the amount of Maillard reaction products generated. This is important since HMF is readily metabolized to 5-sulfooxymethylfurfural (SMF), which can damage DNA though formation of DNA adducts [32]. The consumption of 2 L·day^−1^ of an enteral formula enriched with arginine (the usual intake) and sterilized at 120 °C for 10 min means an HMF intake of 5 mg, which is below the usual HMF intake of the Spanish population [33] and three times lower than those necessary to detect SMF in plasma and DNA adducts [32].

## Figures and Tables

**Table 1:** Loss of fluorescence intensity (%) with OPA at 120 °C. Values are means ± SD, *n* = 3; Different letters within a column indicate significant differences between groups (*p* < 0.05).

Time (min)	Glutamine-Dextrinomaltose	Arginine-Dextrinomaltose
0	0.00 ^a^ ± 0.00	0.00 ^a^ ± 0.00
5	35.53 ^b^ ± 0.72	43.52 ^b^ ± 1.21
10	63.16 ^c^ ± 1.27	74.31 ^c^ ± 0.70
15	77.63 ^d^ ± 0.80	85.91 ^d^ ± 0.73
20	86.05 ^e^ ± 2.12	90.10 ^e^ ± 1.25
25	89.47 ^e^ ± 1.41	91.15 ^e^ ± 2.10
30	90.53 ^e^ ± 1.84	91.48 ^e^ ± 0.78

**Table 2:** 5-Hydroximethylfurfural (HMF) content (mg·L^−1^). Values are means ± SD, *n* = 3; Different letters within a column indicate significant differences between groups (*p* < 0.05).

Time (min)	Glutamine-Dextrinomaltose			Arginine-Dextrinomaltose		
	100 °C	120 °C	140 °C	100 °C	120 °C	140 °C
0	0.04 ^a^ ± 0.01	0.04 ^a^ ± 0.01	0.04 ^a^ ± 0.00	0.04 ^a^ ± 0.01	0.04 ^a^ ± 0.01	0.04 ^a^ ± 0.02
5	0.04 ^a^ ± 0.00	0.04 ^a^ ± 0.02	0.04 ^a^ ± 0.00	0.15 ^b^ ± 0.04	1.35 ^b^ ± 0.09	2.27 ^b^ ± 0.11
10	0.04 ^a^ ± 0.03	0.04 ^a^ ± 0.00	0.26 ^b^ ± 0.05	0.26 ^c^ ± 0.04	2.49 ^c^ ± 0.14	4.19 ^c^ ± 0.31
15	0.04 ^a^ ± 0.02	0.04 ^a^ ± 0.01	0.46 ^c^ ± 0.04	0.35 ^d^ ± 0.07	3.58 ^d^ ± 0.21	5.90 ^d^ ± 0.44
20	0.04 ^a^ ± 0.02	0.19 ^b^ ± 0.02	0.73 ^d^ ± 0.09	0.48 ^e^ ± 0.04	5.07 ^e^ ± 0.28	7.76 ^e^ ± 0.32
25	0.07 ^a^ ± 0.03	0.29 ^c^ ± 0.04	1.00 ^e^ ± 0.11	0.57 ^e^ ± 0.08	6.15 ^f^ ± 0.31	9.59 ^f^ ± 0.49
30	0.14 ^b^ ± 0.03	0.40 ^d^ ± 0.03	1.20 ^e^ ± 0.13	0.78 ^f^ ± 0.06	7.32 ^g^ ± 0.39	11.40 ^g^ ± 0.51

**Table 3:** Furfural content (mg·L^−1^). Values are means ± SD, *n* = 3; Different letters within a column indicate significant differences between groups (*p* < 0.05); n.d. = not detected.

Time (min)	Glutamine-Dextrinomaltose			Arginine-Dextrinomaltose		
	100 °C	120 °C	140 °C	100 °C	120 °C	140 °C
0	n.d.	n.d.	n.d.	n.d.	n.d.	n.d.
5	n.d.	n.d.	n.d.	n.d.	0.32 ^a^ ± 0.04	0.49 ^a^ ± 0.04
10	n.d.	n.d.	0.04 ^a^ ± 0.00	n.d.	0.64 ^b^ ± 0.09	1.17 ^b^ ± 0.11
15	n.d.	n.d.	0.09 ^b^ ± 0.02	n.d.	0.98 ^c^ ± 0.22	1.88 ^c^ ± 0.16
20	n.d.	0.03 ^a^ ± 0.01	0.17 ^c^ ± 0.01	0.02 ^a^ ± 0.01	1.27 ^d^ ± 0.25	2.53 ^d^ ± 0.22
25	n.d.	0.08 ^b^ ± 0.02	0.28 ^d^ ± 0.05	0.04 ^a^ ± 0.01	1.62 ^e^ ± 0.11	3.17 ^e^ ± 0.31
30	0.02 ^a^ ± 0.01	0.16 ^c^ ± 0.04	0.40 ^e^ ± 0.07	0.06 ^a^ ± 0.02	2.89 ^f^ ± 0.32	3.95 ^f^ ± 0.32

**Table 4:** Glucosylisomaltol content (mg·L^−1^) in the model system glutamine-dextrinomaltose. Values are means ± SD, *n* = 3; Different letters within a column indicate significant differences between groups (*p* < 0.05); n.d. = not detected.

Time (min)	100 °C	120 °C	140 °C
0	n.d.	n.d.	n.d.
5	n.d.	n.d.	n.d.
10	n.d.	n.d.	0.50 ^a^ ± 0.04
15	n.d.	0.36 ^a^ ± 0.03	1.22 ^a^ ± 0.10
20	n.d.	0.59 ^a^ ± 0.05	1.91 ^a^ ± 0.13
25	0.26 ^a^ ± 0.03	0.96 ^a^ ± 0.06	2.64 ^a^ ± 0.18
30	0.37 ^a^ ± 0.04	1.35 ^a^ ± 0.10	3.38 ^a^ ± 0.21

**Table 5:** Absorbance at 420 nm. Values are means ± SD, *n* = 3; Different letters within a column indicate significant differences between groups (*p* < 0.05).

Time (min)	Glutamine-Dextrinomaltose			Arginine-Dextrinomaltose		
	100 °C	120 °C	140 °C	100 °C	120 °C	140 °C
5	0.007 ^a^ ± 0.004	0.119 ^a^ ± 0.001	0.886 ^a^ ± 0.064	0.305 ^a^ ± 0.007	1.229 ^a^ ± 0.027	4.555 ^a^ ± 0.096
10	0.014 ^b^ ± 0.001	0.218 ^b^ ± 0.003	1.792 ^b^ ± 0.023	0.581 ^b^ ± 0.002	2.458 ^b^ ± 0.011	6.952 ^b^ ± 0.045
15	0.021 ^c^ ± 0.003	0.326 ^c^ ± 0.004	2.721 ^c^ ± 0.074	0.879 ^c^ ± 0.010	3.676 ^c^ ± 0.058	8.180 ^c^ ± 0.068
20	0.028 ^c^ ± 0.004	0.428 ^d^ ± 0.014	3.600 ^d^ ± 0.014	1.158 ^d^ ± 0.011	4.857 ^d^ ± 0.905	8.850 ^d^ ± 0.008
25	0.035 ^d^ ± 0.003	0.530 ^e^ ± 0.007	4.481 ^e^ ± 0.016	1.452 ^e^ ± 0.011	6.048 ^e^ ± 0.343	8.887 ^d,e^ ± 0.033
30	0.043 ^e^ ± 0.004	0.638 ^f^ ± 0.014	5.402 ^f^ ± 0.014	1.729 ^f^ ± 0.017	7.254 ^f^ ± 0.044	8.892 ^e^ ± 0.014

**Table 6:** Fluorescence intensity evolution. Values are means ± SD, *n* = 3; Different letters within a column indicate significant differences between groups (*p* < 0.05).

Time (min)	Glutamine-Dextrinomaltose			Arginine-Dextrinomaltose		
	100 °C	120 °C	140 °C	100 °C	120 °C	140 °C
0	2.6 ^a^ ± 0.0	2.6 ^a^ ± 0.0	2.6 ^a^ ± 0.0	4.1 ^a^ ± 0.0	4.1 ^a^ ± 0.0	4.1 ^a^ ± 0.0
5	4.2 ^b^ ± 0.1	5.5 ^b^ ± 0.2	10.3 ^b^ ± 0.1	8.8 ^b^ ± 0.1	10.1 ^b^ ± 0.1	20.7 ^b^ ± 0.1
10	6.0 ^c^ ± 0.2	8.5 ^c^ ± 0.2	17.9 ^c^ ± 0.6	13.6 ^c^ ± 0.2	17.1 ^c^ ± 0.1	38.1 ^c^ ± 0.8
15	7.8 ^d^ ± 0.1	11.6 ^d^ ± 0.1	27.0 ^d^ ± 0.6	18.1 ^d^ ± 0.1	24.7 ^d^ ± 0.1	54.6 ^d^ ± 0.2
20	9.6 ^e^ ± 0.2	14.9 ^e^ ± 0.5	36.8 ^e^ ± 0.3	24.2 ^e^ ± 0.3	30.8 ^e^ ± 0.2	72.3 ^e^ ± 0.7
25	11.5 ^f^ ± 0.0	18.1 ^f^ ± 0.1	46.8 ^f^ ± 0.3	29.1 ^f^ ± 0.1	37.4 ^f^ ± 0.2	89.3 ^f^ ± 0.8
30	13.3 ^g^ ± 0.2	21.4 ^g^ ± 0.4	55.9 ^g^ ± 0.5	34.8 ^g^ ± 0.2	45.0 ^g^ ± 0.2	106 ^g^ ± 0.9
